# Dr. Fernand Henrotin

**Published:** 1907-01

**Authors:** 


Dr. Fernand Henrotin, of Chicago, died at his home in that city of myocarditis, December 9, 1906, aged 59 years. He was the son of Dr. Joseph F. Henrotin, also of Chicago. He received his education, academic and medical, in that city, graduating from Rush Medical College in 1868. He served in various capacities from interne to attending surgeon in the Cook County Hospital and was president of the medical board of that institution. He was professor of gynecology at the Chicago Policlinic and at the time of his death was president of that institution. He was a member of various medical bodies and a writer of considerable repute.

